# Exploring home care nurses’ perceived competence and self-efficacy in palliative care delivery: A cross-sectional study

**DOI:** 10.1017/S1478951526102594

**Published:** 2026-05-07

**Authors:** Joanne Ta, Joanne Tay, Kathryn Pfaff

**Affiliations:** Faculty of Nursing, University of Windsor, Windsor, ON, Canada

**Keywords:** Home health nursing, nurses, palliative care, professional competence, self-efficacy

## Abstract

**Background:**

Integration of home-based palliative care (PC) enables patients to receive care at home, fosters family involvement, and reduces healthcare costs. Despite its benefits, nurses report challenges in delivering competent PC, and limited research has explored how home care nurses perceive their own competence and self-efficacy within this context.

**Objectives:**

The study aimed to explore Ontario nurses’ perceived competence and self-efficacy in home-based PC delivery. It also examined the relationship between both constructs, perceived competence and self-efficacy.

**Methods:**

A cross-sectional design was used with 2 validated survey tools: the 10-domain Palliative Care Nursing Self-Competence scale and the 2-domain Palliative Care Self-Efficacy scale. Ontario home care and nursing organizations were contacted to assist with recruitment by disseminating a Qualtrics survey link via mass email to nurses who had provided home-based PC. A minimum of 219 participants was required based on a G*Power analysis. Data were collected over 2 months with 2 reminder emails. Descriptive analysis and Spearman’s rank correlation were conducted to address the research questions.

**Results:**

Seventy-two registered nurses and 38 registered practical nurses reported the highest levels of perceived competence in addressing functional care, while spiritual care emerged as the most challenging domain. Self-efficacy was higher in psychosocial care than in symptom management. A strong positive correlation was found between perceived competence and self-efficacy (*ρ* = .69, *p* <.001), highlighting the interconnected nature of these constructs in home-based PC.

**Significance of results:**

Nurses’ low perceived competence and self-efficacy in spiritual care and symptom management highlight gaps in meeting patients’ holistic care needs. Nurses must be better equipped to manage the psychosocial and spiritual care needs of patients and families. Strengthening training and resources can enhance holistic PC delivery and nurses’ preparedness, thereby supporting nurse retention and the quality and sustainability of home-based PC.

## Introduction

Canada’s aging population, characterized by rising life expectancy and increasing prevalence of complex comorbidities, presents significant challenges to the healthcare system (Steffler et al. 2021). Healthcare institutions are becoming increasingly overwhelmed by workforce shortages and limited bed capacity, particularly in home care, where more Canadians prefer to receive care in their homes (Canadian Cancer Society 2023; Canadian Hospice Palliative Care Association 2024). This is especially important for palliative care (PC) services, where the demand for compassionate, individualized care often exceeds institutional capacity. PC represents a holistic approach focused on improving the quality of life through physical, psychological, spiritual, and social support for both patients and families (World Health Organization 2023). However, as global health systems struggle to meet the demands, only 14% of the 56.8 million people worldwide needing PC receive it (World Health Organization 2020). To address this gap, community-based PC has emerged as an important resource, with home care nurses serving as key providers. However, they face unique challenges in this independent setting that requires a high level of autonomy. Unlike team-based hospital environments, they must adapt to varied home conditions, manage limited resources, and make independent decisions under pressure (Furåker and Nilsson 2012). These conditions, combined with travel time and workload demands, often constrain opportunities for reflection and holistic care (Sawatzky et al. 2021). In such contexts, both competence and self-efficacy become critical, as nurses must rely on their knowledge, skills, and confidence to respond effectively to unpredictable clinical and emotional situations.

### Unpacking the constructs of perceived competence and self-efficacy

Despite distinct definitions, the 2 constructs are often used interchangeably. Perceived competence is one’s belief in their ability to complete a task through possession of knowledge, skills, and attitudes, while self-efficacy is one’s belief in their ability to complete a task under certain circumstances (Bandura 1977; Desbiens and Fillion 2011). The concept of self-efficacy is shaped by 4 sources: one’s own experiences (i.e., past clinical experiences), vicarious experiences (i.e., learning through observing others’ successful performance), verbal persuasion (i.e., encouragement or feedback from others), and emotional arousal (i.e., emotional or physical states that affect confidence). High self-efficacy without sufficient competence can lead to suboptimal and unsafe care, while competence without confidence can lead to ineffective care.

Perceived competence in nursing traditionally focuses on knowledge, skills, and attitude as core attributes that are often developed through practice (Fukada 2018). However, comparatively fewer studies have explored self-efficacy, especially in the context of home care where nurses must possess high self-efficacy to complete tasks under stressful circumstances (Abdal et al. 2015; Iacono et al. 2024; Tomita 2024). This gap could be due to the concept’s complex and abstract nature, making it difficult to measure (Abdal et al. 2015). In addition, much of the existing literature on nursing competence or self-efficacy primarily focuses on hospital settings (Hayter 2016; Shen et al. 2019; Levine 2020; Fadaei et al. 2024), and the context of home care presents unique challenges that may influence the development of these 2 constructs.

### Perceived competence and self-efficacy in home-based PC

While moderate pressure can enhance learning capacity, excessive stress can impair critical thinking and performance (Zhang et al. 2024). These stress-related challenges are particularly relevant in home-based PC where nurses operate with limited access to readily support and face emotionally charged situations. These stressors may contribute to variations in perceived competence and self-efficacy. The literature highlights that nurses demonstrate conflicting competence in pain assessment, communication skills, and advanced care planning, which impacts their self-efficacy in providing effective PC (Furåker and Nilsson 2012; Sawatzky et al. 2021). Lack of knowledge and confidence deters nurses from teaching patients and families about disease progression, leading to skill avoidance that further compromises care (Glajchen and Bookbinder 2001).

There continues to be a prioritization of physical care over spiritual and psychological support within the literature, highlighting the persistent biomedical focus of nursing practice (Furåker and Nilsson 2012; Sawatzky et al. 2021). Even with psychological assessments, nurses often confuse depression with normal grief, reflecting their tendency to rely on physical rather than psychological indicators (Hallford et al. 2012).

In the complex, unpredictable practice of PC, nurses face daily stressors that exacerbate compassion fatigue and burnout and contribute to workforce attrition and staffing shortages (Martens 2009; Canadian Nurses Association 2024). These challenges in communication and holistic care delivery suggest significant variability in both competence and self-efficacy among home care nurses. However, the extent of these variations and how both constructs interact in this setting remain unclear. Therefore, understanding the levels of perceived competence and self-efficacy, as well as their relationship, is important for identifying gaps in nurses’ preparedness and guiding targeted interventions to support holistic, community-based PC.

### Objective and research questions

This study explored Ontario home care nurses’ perceived competence and self-efficacy in delivering PC. The research questions were:

1. What are the perceived levels of competence and self-efficacy among home care nurses in PC delivery?
2. What is the relationship between perceived competence and self-efficacy among home care nurses providing PC?

## Methods

### Study design

A cross-sectional study was conducted using 2 validated survey tools, the Palliative Care Nursing Self-Competence (PCNSC) scale (Desbiens and Fillion 2011) and the Palliative Care Self-Efficacy scale (PCSES) (Phillips et al. 2011), to assess home care nurses’ perceived competence and self-efficacy in PC delivery. This survey was administered through Qualtrics. Home care and professional nursing organizations were contacted to assist in distributing the recruitment flyer and survey link to their nurses through mass email. The survey link was sent to home care nurses in mid-December 2024, and they had 2 months to complete the survey. Through the same means of communication, one reminder email was sent 1 month after survey distribution, and the second reminder was sent 1 week before the end date.

### Recruitment and sample

#### Recruitment

Home care nurses were recruited across 4 home care and 2 professional nursing organizations in Ontario using snowball and convenience sampling methods. We employed snowball sampling because of the specialized nature of palliative home care nursing and the harder-to-reach population within the broader nursing workforce.

#### Sample

Organizations were asked to distribute the study poster and the Qualtrics survey link to their nurses. Inclusion criteria included: (1) registered nurses (RNs) and registered practical nurses (RPNs), (2) currently working as a home care nurse in Ontario, regardless of full-time, part-time, or casual employment, (3) having at least 6 months of nursing experience, and (4) having provided PC in patients’ homes. Exclusion criteria included nurse practitioners, nursing students, and other healthcare providers (HCPs) due to the differences in the scope of practice.

#### Sample size estimation

Using G*Power analysis, an *a priori* sample size of 199 participants was calculated based on an anticipated medium effect size (*r* = 0.20), as reflected in similar studies in PC nursing (Gray et al. 2021; Kang 2021), an alpha level of 0.05, and a desired power of 0.80. After accounting for an estimated 10% attrition rate to allow for potential non-response or incomplete surveys, the target sample size was set at 219 participants.

### Data collection

Data collection ended in March 2025, with a 2-month collection period per organization. The survey consisted of 6 sections: (1) a consent form that participants must accept prior to entering the survey; (2) 4 screening questions to confirm eligibility; (3) 6 demographic questions, such as age, gender, ethnicity, education level, RNs or RPNs, and location of practice; (4) the PCNSC scale (Desbiens and Fillion 2011); (5) the PCSES (Phillips et al. 2011); and (6) 35 general questions developed based on findings from the literature, such as completion of any PC training and education. Participants who did not meet the eligibility criteria were automatically directed to the end of the survey. The estimated completion time was 15–20 minutes. Once completed, they were given a $10 electronic gift card.

### Instruments

#### PCNSC scale

Based on Bandura’s Social Cognitive Theory (1977), Desbiens and Fillion (2011) developed the PCNSC scale to measure nurses’ perceived competence in caring for adult palliative patients across various settings. The 50-item PCNSC scale encompasses 10 domains, each with 5 items: (1) physical needs: pain, (2) physical needs: other symptoms, (3) psychological needs, (4) social needs, (5) spiritual needs, (6) needs related to functional status, (7) ethical and legal issues, (8) interprofessional collaboration and communication, (9) personal and professional issues related to nursing care, and (10) end-of-life care. The scale uses a 0–5 Likert-type response where 0 = not at all capable and 5 = highly capable for each item (Desbiens and Fillion 2011). This instrument was selected due to its comprehensive coverage of PC domains relevant to home care practice and established psychometric properties, with a Cronbach’s alpha of 0.98. Permission was obtained from the researcher to use the tool in this study.

#### PCSES

The PCSES was developed by Phillips et al. (2011) to assess nurses’ self-efficacy in PC delivery. The 12-item scale is separated into 2 domains: psychosocial support (items 1–6) and symptom management (items 7–12). This scale uses a 1–4 Likert-type response where 1 = needs further basic instruction and 4 = confident to perform independently (Phillips et al. 2011). This scale was chosen for its focus on self-efficacy specifically and demonstrated high internal consistency with a Cronbach’s alpha of 0.95. The scale has shown validity across nursing populations with varying levels of experience, from students to practicing nurses (Kim et al. 2020; Zhou et al. 2021; Briese 2022; DeFusco et al. 2023). Permission for its use was obtained from the scale developer.

### Data analysis

Data were exported from Qualtrics to SPSS (version 29.0.2.0) for analysis. Prior to analysis, data were assessed for completeness, outliers, and distributional assumptions. Missing data patterns were examined, and cases with less than 50% completion were excluded using listwise deletion, as these were considered unlikely to provide meaningful responses (Kang 2013). For demographic variables, age had 16 isolated missing values. Data were treated as missing completely at random and excluded from relevant analyses while retaining participants for other variables with complete data.

#### Research question 1

Descriptive analyses (frequencies, percentages, and means) were conducted to summarize participants’ demographic characteristics and overall scores on the PCNSC and PCSES scales. Given the absence of established cut-off scores for both scales and the non-normal distribution of the data, as confirmed by the Shapiro–Wilk test (*p* < .05), medians and interquartile ranges (IQRs) were computed to measure central tendency and dispersion (Desbiens and Fillion 2011; Phillips et al. 2011).

#### Research question 2

Data screening revealed no significant outliers, and normality testing using the Shapiro–Wilk test confirmed non-normal distributions for both PCNSC and PCSES total scores (*p* < .05). Given the ordinal nature of the Likert-type data and the expectation of monotonic rather than linear relationships between variables, Spearman’s rank correlation coefficient was applied using SPSS. This non-parametric approach is less sensitive to outliers and does not assume linearity (Plichta et al. 2012). Furthermore, Cronbach’s alpha was calculated to assess the internal consistency of both instruments.

To examine domain-specific relationships, PCNSC and PCSES domains were conceptually aligned based on established PC frameworks (e.g., Comprehensive Advanced Palliative Care Education). Three domain pairs were constructed: pain management (PCNSC “physical needs: pain” with PCSES item 7), symptom management (PCNSC “physical needs: other symptoms” with PCSES items 8–12), and psychosocial care (combined PCNSC “psychological care” and “social care” domains with PCSES items 1–6). Combining psychological and social care domains was justified based on their conceptual overlap in clinical practice and alignment with the psychosocial support construct in the PCSES. The differing rating systems and construct definitions of both instruments were considered during this alignment to ensure conceptual consistency. The conceptual alignment was intended for interpretive purposes rather than to imply equivalence, and the constructed domain groupings represent post hoc conceptual alignment rather than psychometrically validated subscales. Without factor analysis, the results should be interpreted cautiously. In addition, the total scores of both scales, which differ in their distinct rating systems and construct definitions, were also compared to examine nurses’ overall perceived competence and self-efficacy. These total scores represent the most psychometrically sound indicators with established validity between both constructs.

## Results

### Sample characteristics

The total number of survey distributions was unknown; thus, a true response rate could not be calculated. Of the 184 surveys received, 39 (21.2%) surveys did not meet the criteria, and 35 (19.0%) surveys had less than 50% completed and were excluded, leaving a final sample size of 110.

The majority of the sample identified as female (*n* = 96, 87.3%). Most were between 30 and 39 years old (*n* = 40, 42.6%) and primarily identified as Caucasian/White (*n* = 59, 53.6%). Most were RNs (*n* = 72, 65.5%) with a Bachelor of Science in Nursing (*n* = 61, 55.4%). The largest proportion of participants was from the western region of Ontario (*n* = 27, 24.6%; see Table 1 for demographics).

**Table 1.** Sample characteristics

Variable	*n* (%)
**Age**	
20–29	8 (8.5)
30–39	40 (42.6)
40–49	28 (29.8)
50–59	14 (14.9)
60–69	3 (3.2)
70 or older	1 (1.0)
**Gender**	
Male	12 (10.9)
Female	96 (87.3)
Prefer not to say	2 (1.8)
**Ethnicity**	
White	59 (53.6)
South Asian (e.g., East Indian, Pakistani, Sri Lankan)	12 (10.9)
Chinese	2 (1.8)
Black	20 (18.2)
Filipino	9 (8.2)
Latin American	2 (1.8)
Southeast Asian (e.g., Vietnamese, Cambodian, Laotian, Thai)	2 (1.8)
West Asian (e.g., Iranian, Afghan)	2 (1.8)
Korean	1 (0.9)
Other	1 (0.9)
**Highest education**	
Diploma	40 (36.4)
Bachelor of Science in Nursing	61 (55.4)
Master of Science in Nursing	8 (7.3)
PhD	1 (0.9)
**License**	
RN	72 (65.5)
RPN	38 (34.5)
**Location of practice**	
Northeast Region	12 (10.9)
Northwest Region	4 (3.6)
East Region	22 (20.0)
Central Region	15 (13.6)
Toronto Region	25 (22.7)
West Region	27 (24.6)
Unsure	5 (4.6)

*n* refers to the number of participants who provided data for that variable. Percentages are calculated using the number of valid responses for each variable; missing responses were excluded from the denominator.

### Research question 1

#### Overall PCNSC scale

Overall, nurses reported a high level of perceived competence in PC with a median PCNSC score of 197.5 out of a possible 250.0 (IQR = 174.0–218.0). “Needs related to functional status” had the highest median score among all 10 domains (median = 21.0, IQR = 18.0–24.0). The domain with the lowest perceived competence was “spiritual needs” (median = 17.0, IQR = 15.0–20.0; see Table 2 for more details).

**Table 2.** Nurses’ scores in the PCNSC scale

Domain	Median (IQR)	Summary of competencies assessed
Physical Needs: Pain	20.0 (17.8–23.0)	Pain assessment (verbal/nonverbal), management (pharmacological/non-pharmacological), and monitoring for side effects.
Physical Needs: Other Symptoms	20.0 (17.0–23.0)	Management of nausea, vomiting, constipation, fatigue, dyspnea, and oral hygiene.
Psychological Needs	19.0 (15.0–21.0)	Provide assessment for delirium and depression and offer support to patients and families coping with grief
Social Needs	19.0 (16.0–21.0)	Navigate family dynamics, address cultural needs, provide resources, and supporting communication.
Spiritual Needs	17.0 (15.0–20.0)	Assessing spiritual needs and signs of distress, promote meaning-making and individualized care to align with beliefs.
Needs Related to Functional Status	21.0 (18.0–24.0)	Assistance with activities of daily living, promotion of autonomy, and supporting caregivers.
Ethical & Legal Issues	19.0 (16.0–22.0)	Identifying ethical and legal issues, guide end-of-life decisions, and patient advocacy.
Interprofessional Collaboration & Communication	20.0 (18.0–24.0)	Promote collaboration and communication between providers and families, and conflict resolution.
Personal & Professional Issues Related to Nursing Care	20.0 (18.0–23.0)	Reflecting on own experiences, coping with grief, and communicating about death/dying.
End-of-Life Care	20.0 (18.0–24.0)	Active end-of-life care, recognizing imminent deaths, and respecting cultural and spiritual practices.
Total Score	197.5 (174.0–218.5)	Total score across all 10 domains.

#### Overall PCSES

Nurses’ overall self-efficacy was also high, with a median score of 41.0 out of a possible 48.0 (IQR = 36.0–46.3). Nurses scored slightly higher in the “psychosocial support” domain (median = 21.0, IQR = 18.8–23.0) than in “symptom management” (median = 20.0, IQR = 18.0–24.0; see Table 3 for more details).

**Table 3.** Nurses’ scores in the PCSES

Subscale	Median (IQR)	Summary of competencies assessed
Psychosocial Support	21.0 (18.8–23.0)	Providing emotional support to patients and families, communication regarding death and dying, and offering resources/services.
Symptom Management	20.0 (18.0–24.0)	Management of pain, delirium, dyspnea, and gastrointestinal symptoms, while supporting decision-making capacity.
Total Score	41.0 (36.0–46.3)	Total score across both domains.

### Research question 2

The highest statistically significant positive association was found between nurses’ overall perceived competence and self-efficacy (*ρ = .*69, *p* < .001). Domain-specific analyses showed that “symptom management” had the second highest association (*ρ* = .55, *p* < .001), followed by “psychosocial care” (*ρ* = .54, *p* <.001), and “pain management” (*ρ* = .35, *p* < .001). All correlations were statistically significant at *p* < .001, indicating consistent associations between the constructs (see Table 4 for details).

**Table 4.** Association between nurses’ perceived competence and self-efficacy in PC delivery

			95% Confidence intervals (2-tailed)	
	Spearman’s rho (*ρ*)	Sig. (2-tailed)	Lower	Upper
PCNSC (Total) – PCSES (Total)	.69	<.001	.567	.775
PCNSC: Pain – PCSES: Pain	.35	<.001	.166	.506
PCNSC: Other Symptom Management – PCSES: Other Symptoms	.55	<.001	.402	.673
PCNSC: Psychosocial Care – PCSES: Psychosocial Care	.54	<.001	.387	.663

## Discussion

This study examined Ontario home care nurses’ perceived competence and self-efficacy in delivering PC and explored their relationship. Nurses demonstrated high competence and self-efficacy, though notable gaps remained. They felt most confident in addressing functional needs, least in spiritual care, and more comfortable with psychosocial care than with symptom management. The positive association between the 2 constructs indicates that confidence and skill develop together. These findings suggest that the realities of home care practice influence how nurses develop their skills in providing holistic PC.

While competence (Agoston 2014; Nguyen et al. 2014; Hayter 2016; Levine 2020; Parajuli et al. 2021; Sawatzky et al. 2021) and self-efficacy (Dehghani et al. 2020; Briese 2022; Fadaei et al. 2024) have been studied separately, few studie have examined them together, particularly in palliative home care (Desbiens and Fillion 2011). This gap is critical given the growing demand for home-based care and the unique challenges of delivering PC in the community (World Health Organization 2023). This study addressed the gap by examining a dual perspective on their preparedness for PC and identifying priority areas for tailored education, mentorship, and system-level support.

### Research question 1: understanding the domains

Home care nurses reported overall high levels of both perceived competence and self-efficacy in delivering PC, indicating a generally well-prepared workforce. However, domain-specific results revealed meaningful variation in confidence and capability.

#### An emphasis on functional status

The highest perceived competence was in supporting patients’ functional status, which differs from prior studies where pain or symptom management typically ranked highest (Hayter 2016; Shen et al. 2019; Parajuli et al. 2021). This difference may reflect the unique orientation of home care practice toward maintaining independence and preventing hospitalization, goals that require consistent attention to mobility and daily living support. These tasks are more concrete and frequently encountered in home visits, reinforcing nurses’ perceived clarity and comfort in addressing them.

#### Psychosocial support over symptom management

Despite its centrality to PC, self-efficacy in symptom management was rated lowest by home care nurses. In fact, nurses reported greater self-efficacy in psychosocial care compared to symptom management, which departs from trends observed in hospital settings (Briese 2022). We posit that low confidence in symptom management may stem from both training and system issues. For example, documented delays in obtaining medications and supplies have, in some cases, led to patients being sent to emergency rooms for symptom management (Canadian Broadcasting Corporation 2024) during the timeframe that data were collected in this study.

Greater self-efficacy in psychosocial care may be reflected through the longer-term relationships and emotional connections that home care nurses build with patients and families (Danielsen et al. 2018; Joren et al. 2021). Relational practice remains central to PC, as it emphasizes human connection, presence, compassion, and communication, ensuring that patients’ complex needs are addressed and holistic care is provided (Bertaud et al. 2025). Through these relationships, nurses are repeatedly exposed to managing psychosocial needs, which can enhance their confidence over time. At the same time, it emphasizes the need for provider organizations to strengthen their education and support programs to prevent emotional fatigue among the home care nursing workforce. Even well-trained nurses may struggle to feel effective in home care when there is inadequate structural support. Together, these results highlight the importance of tailoring education and the care system to the realities of community-based palliative nursing.

#### A pervasive gap in spiritual practice

Home care nurses reported the lowest perceived competence in spiritual care, a consistent gap in PC literature (Pesut et al. 2015; Levine 2020; Parajuli et al. 2021; Sawatzky et al. 2021). Despite spiritual care being a core component of PC (World Health Organization 2023), it is often neglected by HCPs and confused with religion (Borneman et al. 2010). This may reflect ambiguity in how spirituality is understood by nurses, limited clinical guidance, and practical challenges such as time constraints during visits (Griffiths et al. 2007; Furåker and Nilsson 2012; Joren et al. 2021; Sawatzky et al. 2021).

In high-pressure environments, spiritual care can be deprioritized without structured training or resources (Rego et al. 2020). A practical and evidence-based resource is the Faith, Importance and Influence, Community, and Address (FICA) Spiritual History tool. This structured tool offers prompts that enable care providers to assess and address patients’ spiritual beliefs, practices, and needs (Borneman et al. 2010). Incorporating resources like FICA into education and clinical training can offer nurses concrete guidance on the abstract concept of spirituality, while also supporting the integration of spiritual assessment into routine visits, ultimately enhancing holistic care (Borneman et al. 2010).

### Research question 2: bridging the constructs of competence and self-efficacy

Our findings reveal a positive association between both perceived competence and self-efficacy among home care nurses, suggesting that confidence and skill develop together in community-based PC. While this study’s findings align with the literature, much of this research has been conducted with nursing students in academic settings (Orkaizagirre-Gómara et al. 2020; Tomita 2024). However, similar patterns regarding more experience and exposure helped increase their confidence, knowledge, and skills (Orkaizagirre-Gómara et al. 2020; Tomita 2024). Our results reflected this relationship within the practical realities of home-based PC, where nurses work independently and with limited support. This finding supports Bandura’s (1977) assertion regarding the interconnectedness of both constructs, highlighting the need to assess both within the context of nursing practice. It suggests that competence and confidence develop together and in the absence of either, even highly skilled nurses may feel uncertain or constrained. Strengthening both constructs in tandem may enhance readiness, quality of care, and nurse retention.

## Implications

### PC education

Both educational interventions and tailored training are essential to sustaining competence and confidence in this unique environment. While integration into undergraduate curricula can help assess and strengthen students’ competence and self-efficacy, limited curricular space may constrain implementation. Alternative strategies such as continuing professional development, mentorship programs, and simulation-based training could offer flexible avenues for reinforcing PC competence beyond formal education (Bandura 1977; Benner 1984; Artino 2012; Yoo and Park 2015; Esteban-Burgos et al. 2024). Organizations should also incorporate PC educators/champions to provide knowledge and resources tailored to local practice environments (Pereira et al. 2021). Finally, nurses should be allocated time and cost coverage for continuing education to ensure their knowledge and practice are up to date (Furåker and Nilsson 2012).

### Enhancing home-based PC practice

Given our findings that symptom management showed the lowest self-efficacy scores while spiritual care had the lowest competence scores, home care organizations should prioritize support in these specific domains. This includes ensuring nurses have readily access to targeted mentorship and resources, along with general supportive infrastructures, including timely interdisciplinary consultations, and adequate time to manage complex care (Glajchen and Bookbinder 2001; Furåker and Nilsson 2012; Sawatzky et al. 2021). Moreover, fostering peer support through mentorship, debriefing sessions, and clinical coaching can sustain self-efficacy and reduce emotional burden (Esteban-Burgos et al. 2024). Together, these approaches can strengthen both competence and confidence, ultimately enhancing the quality and sustainability of PC in the home setting.

### Future research

Future research should further explore the impact of targeted educational programs on the domain-specific gaps identified in this study – low competence in spiritual care and low self-efficacy in symptom management. Longitudinal studies are also needed to examine how both perceived competence and self-efficacy evolve throughout a nurse’s career. Additionally, extending this research into settings such as hospice, long-term care, and rural environments would offer comparative insights and help tailor educational and system-level strategies to diverse care contexts.

## Study limitations

Several methodological limitations should be acknowledged when interpreting these findings. The cross-sectional design limits the ability to draw causal inferences about the relationship between perceived competence and self-efficacy. This study relied on self-reported data, which may introduce self-report biases. The final sample size of 110 participants was lower than the target of 219, reducing statistical power and reflects the anticipated challenge of recruiting from a specialized population. The unknown total number of survey distributions prevented calculation of a true response rate, introducing potential non-response bias through convenience and snowball sampling methods. The study’s geographic restriction to Ontario home care RNs and RPNs limits generalizability to other regions and HCPs. The use of 2 separate validated instruments (PCNSC and PCSES) (Desbiens and Fillion 2011; Phillips et al. 2011) required conceptual alignment of domains for comparative analysis, as the instruments were not originally designed to be used together. The constructed domain groupings (pain management, symptom management, and psychosocial care) have not been psychometrically validated and were developed only for interpretive comparison, not as part of an exploratory statistical analysis.

## Conclusion

In sum, this study highlights critical strengths and gaps in home care nurses’ preparedness for PC, revealing a workforce skilled in practical and physical aspects but less prepared to address spiritual needs, a gap that persists despite its central role in holistic care. The clear association between competence and self-efficacy indicates that improving one without the other is unlikely to produce confident, capable practice. Strengthening both through targeted, context-specific education, experiential learning, and system supports is essential to sustaining a workforce ready to meet the complex and growing demands of PC in the home.

## References

[ref1] Abdal M, Alavi NM and Adib-Hajbaghery M (2015) Clinical self-efficacy in senior nursing students: A mixed- methods study. *Nursing and Midwifery Studies* 4(3), e29143. doi:10.17795/nmsjournal29143. 26576443 PMC4644605

[ref2] Agoston IB (2014) Northern Rural Nurses’ Self-Perceived Competence in Addressing the Spiritual Needs of Patients with Life-Limiting Conditions by Using a Palliative Approach. Master’s thesis. Romania: Department of Nursing, West University of Timisoara.

[ref3] Artino R (2012) Academic self-efficacy: From educational theory to instructional practice. *Perspectives on Medical Education* 1(2), 76–85. doi:10.1007/s40037-012-0012-5. 23316462 PMC3540350

[ref4] Bandura A (1977) Self-efficacy: Toward a unifying theory of behavioral change. *Psychological Review* 84(2), 191–215. doi:10.1037/0033-295X.84.2.191. 847061

[ref5] Benner PE (1984) *From Novice to Expert: Excellence and Power in Clinical Nursing Practice*. Menlo Park, CA: Addison-Wesley.

[ref6] Bertaud S, Wilkinson D and Kelley M (2025) The heart of palliative care is relational: A scoping review of the ethics of care in palliative medicine. *BMC Palliative Care* 24, 150. doi:10.1186/s12904-025-01784-5. 40420093 PMC12105298

[ref7] Borneman T, Ferrel B and Puchalski CM (2010) Evaluation of the FICA Tool for spiritual assessment. *Journal of Pain and Symptom Management* 40(2), 163–173. doi:10.1016/j.jpainsymman.2009.12.019. 20619602

[ref8] Briese PM (2022) Factors Associated with New Hospital Nurses’ Self-Efficacy in Providing Palliative End-Of-Life Care. PhD dissertation. North Dakota: Department of Nursing, University of North Dakota.

[ref9] Canadian Broadcasting Corporation (2024) Medical Supply Delays Disrupting Home Care Across Ontario. *Canadian Broadcasting Corporation*, Available at https://www.cbc.ca/news/canada/toronto/supple-delays-ontario-homecare-1.7360120 (accessed 22 October 2024).

[ref10] Canadian Cancer Society (2023) Analyzing Hospice Palliative Care Across Canada. Available at https://cancer.ca/-/media/files/about-us/media-releases/2023/palliative-care-report/adv23163palliative-care-report85x11en04.pdf (accessed 1 June 2025).

[ref11] Canadian Hospice Palliative Care Association (2024) Milestones in Hospice Palliative Care. Available at https://www.chpca.ca/education/milestones-in-hospice-palliative-care/ (accessed 1 June 2025).

[ref12] Canadian Nurses Association (2024) Latest Health Workforce Data Confirms CNA’s Predictions of Critical Nursing Shortages. Available at https://www.cna-aiic.ca/en/blogs/cn-content/2024/02/29/latest-health-workforce-data-confirms-cnas (accessed 1 June 2025).

[ref13] Danielsen BV, Sand AM, Rosland JH, et al. (2018) Experiences and challenges of home care nurses and general practitioners in home-based palliative care–a qualitative study. *BMC Palliative Care* 17(95), 1–18. doi:10.1186/s12904-018-0350-0. PMC6052702 30021583

[ref14] DeFusco C, Lewis A and Cohn T (2023) Improving critical care nurses’ perceived self-efficacy in providing palliative care: A quasi-experimental study. *American Journal of Hospice & Palliative Medicine* 40(2), 117–121. doi:10.1177/10499091221094313. 35513023

[ref15] Dehghani F, Barkhordari-Sharifabad M, Sedaghati-kasbakhi M, et al. (2020) Effect of palliative care training on perceived self-efficacy of the nurses. *BMC Palliative Care* 19(1), 63. doi:10.1186/s12904-020-00567-4. 32366232 PMC7199299

[ref16] Desbiens JF and Fillion L (2011) Development of the palliative care nursing self-competence scale. *Journal of Hospice & Palliative Nursing* 13(4), 230–241. doi:10.1097/NJH.0b013e318213d300.

[ref17] Esteban-Burgos AA, Moya-Carramolino J, Vinuesa-Box M, et al. (2024) Clinical simulation in palliative care for undergraduate nursing students: A randomized clinical trial and complementary qualitative study. *Healthcare* 12(4), 421. doi:10.3390/healthcare12040421. 38391797 PMC10888368

[ref18] Fadaei S, Forouzi MA, Miyashita M, et al. (2024) Palliative care knowledge and self-efficacy: A comparative study between intensive care units and general units nurses. *BMC Palliative Care* 23, 246. doi:10.1186/s12904-024-01580-7. 39438875 PMC11494781

[ref19] Fukada M (2018) Nursing competency: Definition, structure and development. *Yonago Acta Medica* 61(1), 1–7. doi:10.33160/yam.2018.03.001. 29599616 PMC5871720

[ref20] Furåker C and Nilsson A (2012) Registered nurses’ views on competencies in home care. *Home Health Care Management & Practice* 24(5), 221–227. doi:10.1177/1084822312439579.

[ref21] Glajchen M and Bookbinder M (2001) Knowledge and perceived competence of home care nurses in pain management: A national survey. *Journal of Pain and Symptom Management* 21(4), 307–316. doi:10.1016/S0885-3924(01)00247-0. 11312045

[ref22] Gray JR, Grove SK and Sutherland S (2021) *The Practice of Nursing Research: Appraisal, Synthesis, and Generation of Evidence*, 9th Edn. St. Louis, Missouri: Elsevier.

[ref23] Griffiths J, Ewing G, Rogers M, et al. (2007) Supporting cancer patients with palliative care needs: District nurses’ role perceptions. *Cancer Nursing* 30(2), 156–162. doi:10.1097/01.NCC.0000265013.63547.4a. 17413782

[ref24] Hallford DJ, McCabe MP, Mellor D, et al. (2012) Depression in palliative care settings: The need for training for nurses and other health professionals to improve patients’ pathways to care. *Nurse Education Today* 32(5), 556–560. doi:10.1016/j.nedt.2011.07.011. 21862185

[ref25] Hayter CR (2016) Acute Care Nurses’ Self-Reported Competence in Palliative Care. Master’s thesis, Department of Nursing, Montana State University, Bozeman.

[ref26] Iacono CL, Amodio E, Vella G, et al. (2024) Self-perceived competencies and attitudes on palliative care in undergraduate nursing students: A multicenter descriptive study. *Nursing Reports* 14(3), 2550–2564. doi:10.3390/nursrep14030188. 39330742 PMC11435199

[ref27] Joren CY, Veer AJE, Groot K, et al. (2021) Home care nurses more positive about the palliative care that is provided and their own competence than hospital nurses: A nationwide survey. *BMC Palliative Care* 20(170). doi:10.1186/s12904-021-00866-4. PMC8552607 34711219

[ref28] Kang H (2013) The prevention and handling of the missing data. *Korean Journal of Anesthesiology* 64(5), 402–406. doi:10.4097/kjae.2013.64.5.402. 23741561 PMC3668100

[ref29] Kang H (2021) Sample size determination and power analysis using the G*Power software. *Journal of Educational Evaluation for Health Professions* 18, 17. doi:10.3352/jeehp.2021.18.17. 34325496 PMC8441096

[ref30] Kim JS, Kim J and Gelegjamts D (2020) Knowledge, attitude and self-efficacy towards palliative care among nurses in Mongolia: A cross-sectional descriptive study. *PLOS ONE* 15(7), 1–15. doi:10.1371/journal.pone.0236390. PMC7377484 32702007

[ref31] Levine ME (2020) Palliative Care Self-Competence: An Examination of Registered Nurses in the Acute Care Hospital During the Covid-19 Pandemic. PhD dissertation, Department of Nursing, William Paterson University, New Jersey.

[ref32] Martens ML (2009) A comparison of stress factors in home and inpatient hospice nurses. *Journal of Hospice & Palliative Nursing* 11(3), 144–153. doi:10.1097/NJH.0b013e3181a1ac87.

[ref33] Nguyen LT, Yates P and Osborne Y (2014) Palliative care knowledge, attitudes and perceived self-competence of nurses working in Vietnam. *International Journal of Palliative Nursing* 20(9), 448–456. doi:10.12968/ijpn.2014.20.9.448. 25250550

[ref34] Orkaizagirre-Gómara A, Miguel MS, Elguea JO, et al. (2020) Testing general self-efficacy, perceived competence, resilience, and stress among nursing students: An integrator evaluation. *Nursing & Health Sciences* 22,529–538. doi:10.1111/nhs.12689. 32128979

[ref35] Parajuli J, Hupcey JE, Kitko L, et al. (2021) Palliative care: Oncology nurses’ confidence in provision to patients with cancer. *Clinical Journal of Oncology Nursing* 25(4), 449–455. doi:10.1188/21.CJON.449-455. 34269349

[ref36] Pereira J, Giddings G, Sauls R, et al. (2021) Navigating design options for large-scale interprofessional continuing palliative care education: Pallium Canada’s experience. *Palliative Medicine Reports* 2(1), 226–236. doi:10.1089/pmr.2021.0023. 34927146 PMC8675227

[ref37] Pesut B, McLean T, Reimer-Kirkham S, et al. (2015) Educating registered nursing and healthcare assistant students in community-based supportive care of older adults: A mixed methods study. *Nurse Education Today* 35(9), e90–e96. doi:10.1016/j.nedt.2015.07.015. 26249645

[ref38] Phillips J, Salamonson Y and Davidson PM (2011) An instrument to assess nurses’ and care assistants’ self-efficacy to provide a palliative approach to older people in residential aged care: A validation study. *International Journal of Nursing Studies* 48(9), 1096–1100. doi:10.1016/j.ijnurstu.2011.02.015. 21377679

[ref39] Plichta SB, Kelvin EA and Munro BH (2012) *Munro’s Statistical Methods for Health Care Research*, 6th Edn. Philadelphia: Wolters Kluwer Health/Lippincott Williams and Wilkins.

[ref40] Rego F, Gonçalves F, Moutinho S, et al. (2020) The influence of spirituality on decision-making in palliative care outpatients: A cross-sectional study. *BMC Palliative Care* 19(1), 22. doi:10.1186/s12904-020-0525-3. 32085765 PMC7035674

[ref41] Sawatzky R, Roberts D, Russell L, et al. (2021) Self-perceived competence of nurses and care aides providing a palliative approach in home, hospital, and residential care settings: A cross-sectional survey. *Canadian Journal of Nursing Research* 53(1), 64–77. doi:10.1177/0844562119881043. 31645110

[ref42] Shen Y, Nilmanat K and Promnoi C (2019) Palliative care nursing competence of Chinese oncology nurses and its related factors. *Journal of Hospice & Palliative Nursing* 21(5), 404–411. doi:10.1097/NJH.0000000000000581. 31166301

[ref43] Steffler M, Li Y, Weir S, et al. (2021) Trends in prevalence of chronic disease and multimorbidity in Ontario, Canada. *CMAJ: Canadian Medical Association Journal* 193(8), e270–e277. doi:10.1503/cmaj.201473. 33619067 PMC8034347

[ref44] Tomita R (2024) The relationship between general self-efficacy and nursing practice competence for second-year nurses: Empirical quantitative research. *NursingOpen* 11(7), 2–10. doi:10.1002/nop2.2233. PMC11222662 38961662

[ref45] World Health Organization (2020) Palliative Care. Available at https://www.who.int/news-room/fact-sheets/detail/palliative-care (accessed 1 June 2025).

[ref46] World Health Organization (2023) Palliative Care. Available at https://www.who.int/europe/news-room/fact-sheets/item/palliative-care (accessed 1 June 2025).

[ref47] Yoo MS and Park HR (2015) Effects of case-based learning on communication skills, problem-solving ability, and learning motivation in nursing students. *Nursing & Health Sciences* 17(2), 166–172. doi:10.1111/nhs.12151. 24889910

[ref48] Zhang X, Guo L, Wang Y, et al. (2024) Effect of stress on study skills self-efficacy in Nursing students: The chain mediating role of general self-efficacy and self-directed learning. *BMC Nursing* 23, 839. doi:10.1186/s12912-024-02500-z. 39548541 PMC11568635

[ref49] Zhou Y, Li Q and Zhang W (2021) Undergraduate nursing students’ knowledge, attitudes and self‐efficacy regarding palliative care in China: A descriptive correlational study. *Nursing Open* 8(1), 343–353. doi:10.1002/nop2.635. 33318842 PMC7729553

